# Removal of Copper (II), Zinc (II), Cobalt (II), and Nickel (II) Ions by PIMs Doped 2-Alkylimidazoles

**DOI:** 10.3390/membranes12010016

**Published:** 2021-12-23

**Authors:** Elzbieta Radzyminska-Lenarcik, Kamila Maslowska, Wlodzimierz Urbaniak

**Affiliations:** 1 Faculty of Chemical Technology and Engineering, Bygdoszcz University of Science and Technology, 85-796 Bydgoszcz, Poland; 2 Faculty of Chemistry, Adam Mickiewicz University, 61-712 Poznan, Poland; kamila.maslowska@amu.edu.pl (K.M.); Wlodzimierz.Urbaniak@amu.edu.pl (W.U.)

**Keywords:** polymer inclusion membrane, non-ferrous metal separation, copper, zinc, cobalt, nickel

## Abstract

Polymer inclusion membranes (PIMs) are an attractive approach to the separation of metals from an aqueous solution. This study is concerned with the use of 2-alkylimidazoles (alkyl = methyl, ethyl, propyl, butyl) as ion carriers in PIMs. It investigates the separation of copper (II), zinc (II), cobalt (II), and nickel (II) from aqueous solutions with the use of polymer inclusion membranes. PIMs are formed by casting a solution containing a carrier (extractant), a plasticizer (o-NPPE), and a base polymer such as cellulose triacetate (CTA) to form a thin, flexible, and stable film. The topics discussed include transport parameters, such as the type of carrier, initial fluxes, separation coefficients of copper in relation to other metals, as well as transport recovery of metal ions. The membrane was characterized using AFM and SEM to obtain information on its composition.

## 1. Introduction

The need for a more specific system for the recovery of non-ferrous metal, one that is more practical from both an economic and ecological standpoint, has led to the development of a new separation technique [1,2,3,4].

In recent years, it has been shown that membrane techniques have advantages over traditional metal compound removal and separation methods [5]. The use of liquid membranes—particularly polymer inclusion membranes (PIMs)—is especially distinguished in this respect [6,7]. PIMs have been proven to be a better alternative than ion exchange and solvent extraction methods. The advantage of using them is that the separation process is carried out in one step (unit process) and no toxic organic solvents are used because in PIMs both the extraction and back extraction processes occur simultaneously. For example, PIMs are successfully used in the recovery and separation of such metals as Cu [8,9], Co [10,11], Ni [10,12,13], Zn [12,14,15,16], Pb [12,17,18], Cd [10,12,19], Hg [20,21], Cr(III) [12,22], Cr(VI) [23], Mn [12], As [12,24], Fe [12], U [12,25], Ag [26,27], Au [28], as well as platinum group metals [29,30], lanthanides, and actinides [12,31,32].

A PIM is made from a base polymer, plasticizer, and carrier of metal ions. The polymer plays a key role in providing the membrane with mechanical strength, and its properties greatly affect the membrane’s permeability and durability. The solutions most often used as polymer matrices are CTA (cellulose triacetate) and PVC (polyvinyl chloride) [1,5,6,33]. The role of the plasticizer is to increase the flexibility and mechanical strength of the polymer matrix by penetrating between its polymer molecules and reducing the strength of the intermolecular forces, thereby increasing the distance between the polymer molecules. Some of the most commonly used plasticizers include o-nitrophenyl octyl and o-nitrophenyl pentyl ethers [6,34]. In principle, carriers used in PIMs are the same organic compounds as those used as extractants in solvent extraction [2,3,5,6,12]. It is crucial for a carrier to be stable on the feed phase side, as well as to readily decay at the membrane/receiving phase interface. Furthermore, a good carrier should be inexpensive, non-toxic, and soluble in the membrane [5,6,34,35]. The carrier’s primary function is to facilitate the transport of separated ions across the membrane. The following groups of carriers can be distinguished based on the chemical properties and the nature of interactions with metal ions:

- acidic carriers—capable of exchanging a proton for a metal ion; the most commonly used transporters of this group include organophosphorus acids (e.g., D2EHPA, Cyanex 272, Cyanex 303) [25,36,37,38], as well as hydroxyoximes (e.g., LIX-84 I) [39,40,41];
- alkaline carriers—organic compounds whose nature corresponds to Lewis bases; they form ionic pairs with metal ions. This group includes quaternary ammonium [42,43,44] and phosphonium salts [33,43], tertiary amines [44,45], pyridine and pyridine derivatives [6,46,47], and alkyl imidazole derivatives [8,27,48];
- inert carriers—capable of forming an inert complex with metal ions in the organic phase by replacing the water molecules in the metal aqua complex with their own molecules, which are more lyophilic. The group of these transporters includes such compounds as phosphoric acid esters [49,50] and phosphinic acid esters [51].

While commercial carriers of metal cations commonly used in the membrane technique enable the effective separation of ions, their selectivity is rather poor. Therefore, new complexing reagents are sought—ones that can selectively separate metal ions from aqueous solutions.

This paper presents a laboratory experiment in which polymer inclusion membranes doped with 2-alkylimidazole (alkyl = methyl, ethyl, propyl, butyl) were tested in order to assess their efficiency in the separation of copper, zinc, cobalt, and nickel ions from their equimolar mixtures. These metals are important for the development of the economy, especially for the development of modern technologies. The demand for them is still growing. In our previous work, alkyl imidazole derivatives were used as carriers in PIMs to separate non-ferrous metal ions [48]. It has been observed that the ion separation efficiency is affected by both the hydrophobic effect, which depends on the length of the alkyl substituent bound to the imidazole molecule [8,52], and the steric effect (steric hindrance) induced by the methyl substituent located in the imidazole molecule at position 2 or 4, i.e., at the donor nitrogen atom [53,54]. In this study, the influence of the length of the alkyl substituent at position 2 in the alkylimidazole molecule was investigated—i.e., the influence of the growth of the hindrance on the separation efficiency of copper, zinc, cobalt, and nickel ions.

## 2. Materials and Methods

### 2.1. Materials

All the reagents used were of analytical grade. Cellulose triacetate, 2-nitrophenyl pentyl ether (NPPE,) and dichloromethane were obtained from Fluka. All the stock solutions were prepared by dissolving the salts in distilled water. CoCl_2_ 6H_2_O, NiCl_2_ 6H_2_O, ZnCl_2_, and CuCl_2_ 2H_2_O were purchased from POCh, Gliwice, Poland. The 2-alkylimidazole (alkyl = methyl, ethyl, propyl, butyl) were synthesized by A. Skrzypczak (Poznan University of Technology, Institute of Chemical Technology and Engineering) according to the procedure described in [55]. Table 1 shows certain physical and chemical properties of the 2-alkylimidazoles used in this study.

### 2.2. Procedure

PIMs were made from CTA, NPPE, and 2-alkylimidazole and were prepared according to the procedure reported in the previous paper [8,52,53,54]. As shown in papers [8,48,52,53,54], the composition of a membrane containing alkylimidazoles as a carrier is optimal when such membrane contains 2.6 cm^3^ of NPPE per 1 g CTA and 1.0 mol/dm^3^ of alkylimidazole calculated on plasticizer. 

Transport experiments were carried out at a temperature of 20 ± 0.2 °C, according to the procedure described in our previous paper [8,52,53,54]. The initial concentration (C_0_) of each metal ion in the feed phase was 10^−3^ mol/dm^3^. The feed aqueous phase was an aqueous solution with a pH of 6.0 (tetramethylammonium hydroxide) while the receiving phase was distilled water. In the feed phase, the metal concentration (C_t_) was determined at appropriate time intervals using the atomic absorption spectroscopy method (AAS Spectrometer, AAS240FS, Agilent, Santa Clara, CA, USA). 

The kinetics of the transport across PIMs was described as a first-order reaction in metal ion concentration (ln(C_0_/C_t_) = −*k*t; where *k* is the rate constant, and t is the time of transport). The *k* values were calculated from the plots of ln(C_0_/C_t_) vs. time. As expected, the relationship of ln(C_0_/C_t_) vs. time was linear. The initial fluxes (J_o_) were determined as J_o_ = (−*k*V/AC_0_), where V is the volume of the feed phase and A is the area of the membrane.

## 3. Results and Discussion

### 3.1. Membrane Characterization

**Figure 1:**
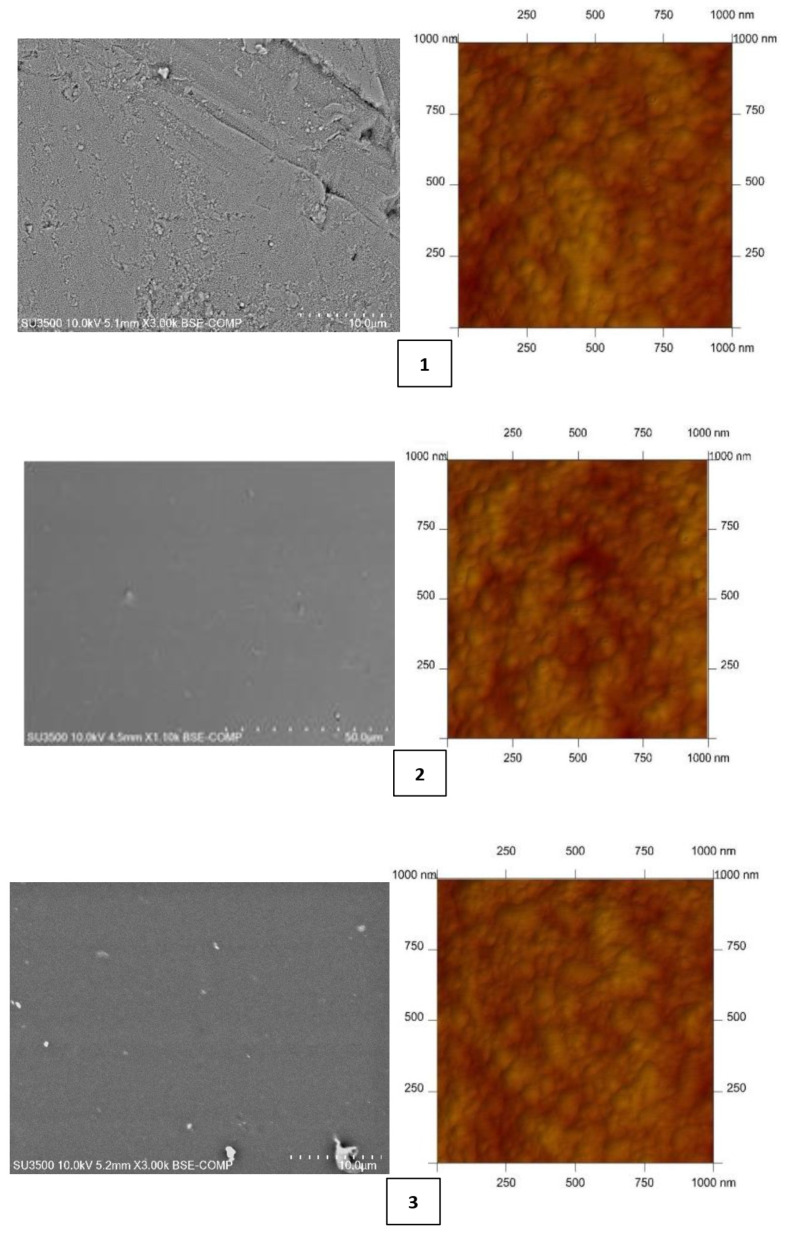

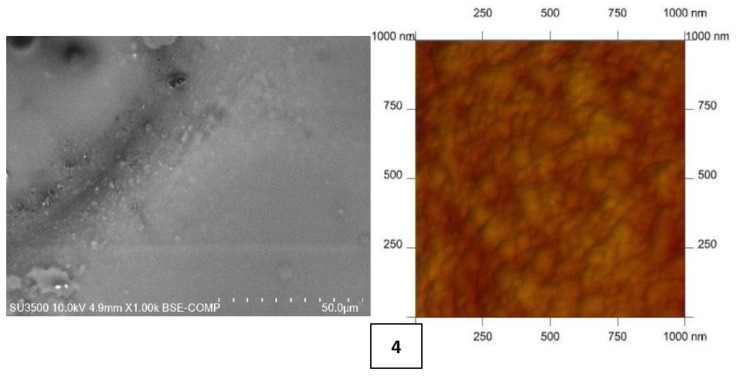
SEM and 2D AFM images of PIMs doped 2-methylimidazole (**1**), 2-ethylimidazole (**2**), 2-propylimidazole (**3**), and 2-butylimidazole (**4**).

As shown in numerous studies [10,15,48,50,51,52,53,54], the membrane’s physicochemical properties affect the selectivity of metal ion transport. Both scanning electron microscopy (SEM) (Hitachi SU3500 SEM/EDS Energy-Dispersive Spectroscopy Hitachi, Tokyo, Japan) and atomic-force MultiMode Scanning Probe Microscope IIIa (AFM) (Digital Instruments Veeco Metrology Group, Santa Barbara, CA, USA)—utilized in air and at room temperature—were used to characterize the PIM surfaces. Figure 1 shows AFM and SEM images of PIMs with carriers **1**–**4**.

Both the SEM and AFM images in Figure 1 indicate that the carrier distribution in the investigated membranes is homogeneous throughout the entire surface after the solvent’s evaporation. 

The average thickness of all membranes was measured using a Panametrics^®^ Magna-Mike^®^ 8500 (San Diego, CA, USA) manual precision thickness gauge. Mean roughness values of the membrane were calculated using AFM. Both the average thickness and roughness values are summarized in Table 2.

CTA-NPPE-2-alkylimidazole (**1**–**4**) membranes form thin films with a thickness of 26–30 µm. Many authors [57,58,59,60,61] have shown that microstructure of PIMs affects the transport of metal ions. Their roughness of PIMs **1**–**4** varies from 3.55 to 4.47 nm. These values are comparable to the values obtained for PIMs with other homologous series of alkylimidazoles (roughness of 2.2–7.2 nm) [48,57,58,59]; however, it is less than that of the CTA membrane obtained by Tor et al., which amounted to 14 nm [22]. The roughness values are also comparable with a CTA membrane doped with a thioazocrown derivative imidazole (a roughness of 3.3–5.3 nm) reported by Ulewicz [60] and with a membrane containing D2EHPA prepared by Salazar-Alvarez [61], whose roughness was 4.6 nm.

### 3.2. Membrane Transport

**Figure 2:**
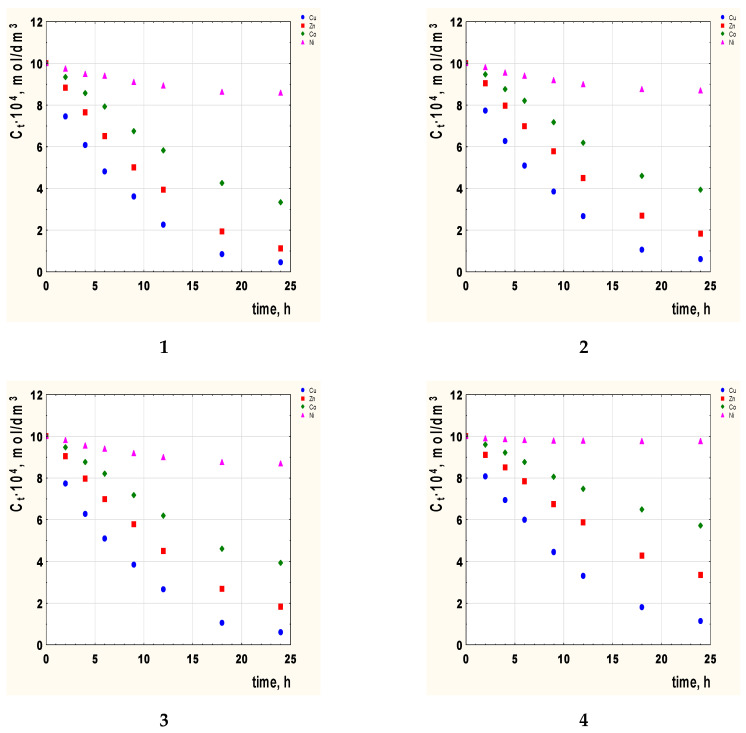
The concentration Cu(II), Zn(II), Co(II), and Ni(II) ions vs. time for PIMs with 2-alkylimidazole as the carrier. 2-methylimidazole (**1**), 2-ethylimidazole (**2**), 2-propylimidazole (**3**), and 2-butylimidazole (**4**). Membrane: 2.6 cm^3^ o-NPPE/1 g CTA and 1.0 mol/dm^3^.

The transport of Co(II), Cu(II), Ni(II), and Zn(II) ions through PIMs from an equimolar mixture was studied using 2-alkylimidazole (alkyl = methyl, ethyl, propyl, butyl) as a carrier. Figure 2 shows changes in the ion concentration of the studied metals in the feed phase during this process.

On the basis of Figure 2, it can be seen that regardless of the type of carrier used in the membrane, the changes in the concentrations of the tested metal ions can be arranged in the order Cu(II) > Zn(II) > Co(II) > Ni(II).

The optimal transport time is 24 h. After this time, the state of equilibrium is established and a further extension of the time does not change the concentrations of metals.

Table 3 shows the initial flux values (J_0_) of the studied metal ions, depending on the type of carrier used.

The data in Table 3 show that the rate of transport of the studied metal ions decreases in the series Cu(II) > Zn(II) > Co(II) > Ni(II). As the length of the alkyl chain in the carrier molecule increases, the initial fluxes of all ions decrease. This phenomenon may be explained by kinetic factors in the formation of complexes of the studied metals at the membrane/feed phase interface. Ni(II) ion transport is particularly low, and in the case of membrane **4,** it is completely inhibited. Ni(II) ions are virtually never transported across membranes containing 2-alkylimidazoles as carriers; they remain in the feed phase.

### 3.3. Complexation Mechanism

The transport process is similar for all carrier types, especially alkyl imidazole derivatives [8,27,48,52,53,54]. The alkyl substituent at position 2 hinders the formation of all metal complexes, as evidenced by the lower values of the stability constants (log β) exhibited by 2-alkylimidazole complexes compared to 1-alkylimidazoles (Table 4).

**Figure 3:**
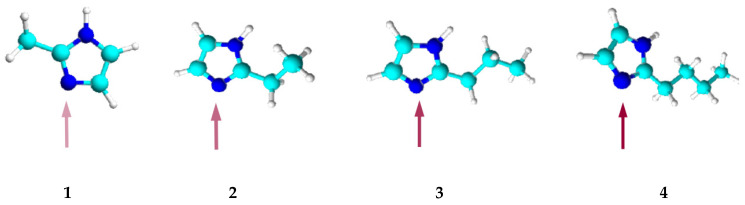
Models of the 2-alkylimidazole molecule, 2-methylimidazole (**1**), 2-ethylimidazole (**2**), 2-propylimidazole (**3**), and 2-butylimidazole (**4**).

The alkyl substituent located at position 2 in the alkylimidazole molecule is a steric hindrance to the binding of metal ions as it blocks access to the donor nitrogen atom. The longer the substituent, the more difficult the complexation reaction (Figure 3). 

The steric effect represents a particular hindrance to the formation of Ni(II) as well as Zn(II) and Co(II) octahedral complexes. It is difficult for Ni(II) ions to bind with 2-alkylimidazoles, and in the case of 2-butylimidazole, a binding reaction does not occur at all (Table 4). Ni(II) ions mostly form 6-coordination complexes, because they have a rigid octahedral structure that is hard to deform. On the other hand, apart from the 6-coordinated ones, Zn(II) and Co(II) ions can also form tetrahedral complexes, which are more easily transferred by PIMs due to their smaller volume. Among the four cations studied, coordination sphere deformation is highly likely to occur only in the case of Cu(II) ions—due to the Jahn–Teller effect [70]—making the transport of such ions the easiest.

Due to differences in the complexation reaction using 2-alkylimidazoles, it is possible to selectively separate Cu(II) ions, particularly from Ni(II) ions (Table 3).

**Figure 4:**
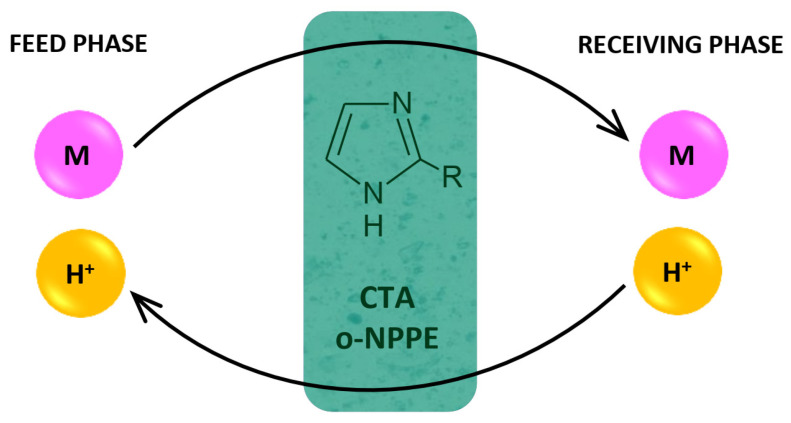
Schematic transport of metal ions across PIM doped 2-alkylimidazoles.

Figure 4 presents the proposed mechanism of the transport of M(II) ions across PIMs.

In the transport mechanism shown in Figure 4, the complexation reaction takes place between the metal ion and 2-alkylimidazoles (L). Complex ions are formed in the membrane:

for Cu(II), Zn(II) Co(II)       M^2+^ + 4 L ↔ [ML_4_]^2+^

for Ni(II)                               M^2+^ + 6 L ↔ [ML_6_]^2+^

The complex ion is then transferred across the membrane towards the receiving phase. Complex ions dissociate at the interface between the membrane and the receiving phase.

At the same time, the proton ions are transported from the receiving phase in the same way towards the feed phase.

### 3.4. Diffusion of Metal Ions across PIMs

**Figure 5:**
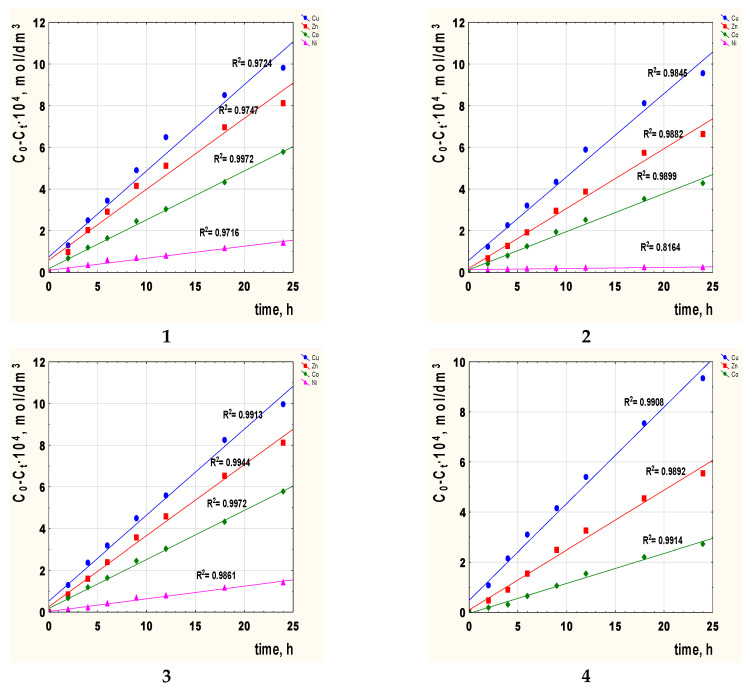
The relationship C_0_-C_t_ vs. time of metal ions transport across PIMs with 2-alkylimidazole **1**–**4** as the carrier; 2-methylimidazole (**1**), 2-ethylimidazole (**2**), 2-propylimidazole (**3**), and 2-butylimidazole (**4**).

The next step consisted in calculating the diffusion coefficient (D_o_) of the metal complex across the membrane doped with 2-alkylimidazole. Figure 5 shows the correlation graphs C_0_-C_t_ vs. time of transport for metal ions with the carriers **1**–**4** across PIMs. 

The diffusion coefficient of each metal ion was calculated, substituting D_o_ = d_o_/Δ_o_, where d_o_ is the thickness of the membrane (Table 2), and Δ_o_ could be evaluated based on the angle of inclination of the lines presented in Figure 5. Table 5 shows the obtained values of diffusion coefficients.

The value of the diffusion coefficient of M(II)-carrier species of 2.38 × 10^−8^–4.13 × 10^−13^ cm^2^/s (Table 5) is smaller than the value of 1.5 × 10^−7^ cm^2^/s reported for the Pb(II) complex with the D2EHPA in PIM by Salazar-Alvarez [61] and is within the range of 10^−8^–10^−13^ cm^2^/s, which indicates that the rate-determining step in the transport of metal ions is the passage across the membrane.

### 3.5. Transport Recovery

**Figure 6:**
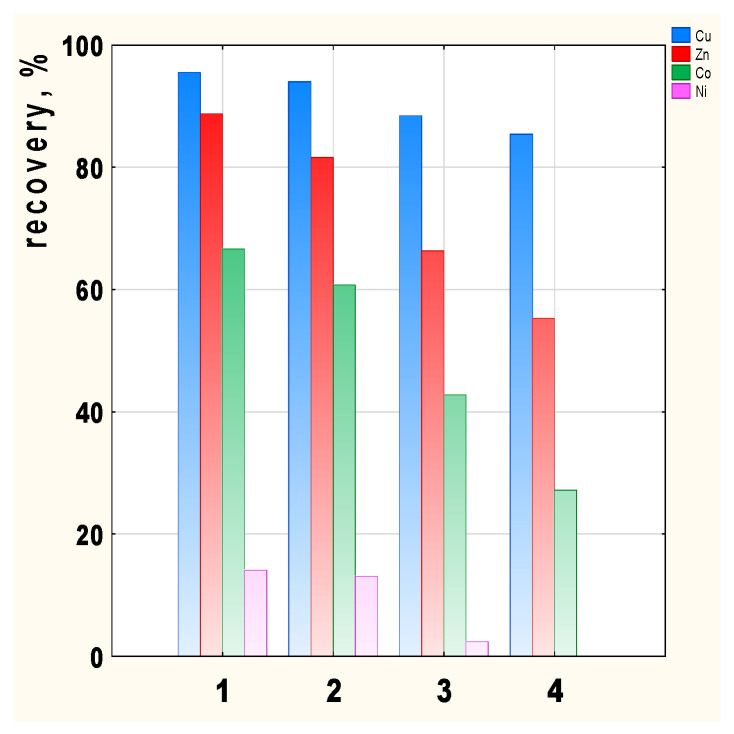
Transport recovery of Cu(II), Zn(II), Co(II), and Ni(II) ions after 24 h depending on the carrier used; 2-methylimidazole (**1**), 2-ethylimidazole (**2**), 2-propylimidazole (**3**), and 2-butylimidazole (**4**).

The transport recovery of Cu(II), Zn(II), Co(II), and Ni(II) ions from their equimolar solutions as a result of transport by PIMs doped with 2-alkylimidazoles **1**–**4** is shown in Figure 6.

Metal recovery depends on the carrier used in the membrane, with the highest recovery values achieved in the case of 2-methyl- (**1**) and 2-ethylimidazole (**2**) (Figure 6). Cu(II) recovery is the highest, ranging from 95.5 (membrane **1**) to 85.4% (membrane **4**) depending on the membrane used. Zn(II) and Co(II) recovery clearly decrease for carriers **3** and **4**. There is little transport of Ni(II) ions across all the membranes tested, hence the Ni(II) recovery is 14% for membrane **1**. In the case of the 2-butylimidazole membrane (**4**), Ni(II) ions remain in the feed phase.

### 3.6. Comparison of the Results with Previously Tested Alkyl Imidazole Derivatives

Table 6 shows the copper (II) separation coefficients in relation to Zn(II), Co(II), and Ni(II) from their equimolar solutions after a 24 h transport across PIMs with alkylimidazoles (1-alkylimidazoles, 1-alkyl-2-methylimidazoles, and 1-alkyl-4-methylimidazoles).

In the case of PIM with 2-alkylimidazoles, the copper (II) separation coefficients in relation to Zn(II) and Co(II) are lower (Table 6). However, these membranes efficiently separate Ni(II) ions from Cu(II)-Zn(II)-Co(II)-Ni(II) mixture.

Table 7 contains the values of Cu(II) recovery in transport across PIMs doped with alkylimidazoles. 

The data in Table 7 indicate that copper recovery after a 24-hour process of transport using alkylimidazole derivatives as carriers are high, which proves the efficiency of alkylimidazoles in the process of separation of Cu(II) ions. The highest Cu(II) recovery factors were achieved when using 1-hexylimidazole [48].

PIMs with 2-alkylimidazoles can be used both for the recovery of Cu(II) from Cu(II)-Zn(II)-Co(II)-Ni(II) mixtures, as well as for the separation of Cu (II) from Ni (II).

## 4. Conclusions

In the process of transport across polymer inclusion membranes using 2-alkylimidazole as a carrier, the rate of transport of metal ions studied decreases in the series Cu(II) > Zn(II) > Co(II) > Ni(II). The presence of a substituent at position 2 affects the value of all parameters characterizing transport. As the length of this substituent increases, the following changes can be observed:

- An increase in the initial fluxes of Cu(II) ions and a decrease in the initial fluxes of Zn(II), Co(II), and Ni(II) ions, up to a complete disappearance of the flux of Ni(II) ions when using 2-butylimidazole,
- An increase in the separation coefficients of Cu(II) ions relative to those of other metals. The highest separation coefficient values for Cu(II)/Zn(II) and Cu(II)/Co(II) are 2.6, 6.9 (membrane **4**), and in the case of Cu(II)/Ni(II), 182 (membrane **3**), respectively.
- A decrease in the recovery efficiency of each metal. Cu(II), Zn(II), Co(II), and Ni(II) recovery is the highest for the membrane with carrier **1** (2-methylimidazole) and amounts to 95.5%, 88.8%, 66.7%, and 14.1%, respectively.

The differences in ion transport and separation efficiency are caused by differences in the complexation reaction of Cu(II), Zn(II), Co(II), and Ni(II) with 2-alkylimidazoles.

## Figures and Tables

**Table 1:** The general formula of the 2-alkylimidazoles used in the tests and some of their physicochemical properties.

	R = alkyl	No	Dissociation Constants, pK_a_ [56]	Melting Point, °C	Boiling Point, °C
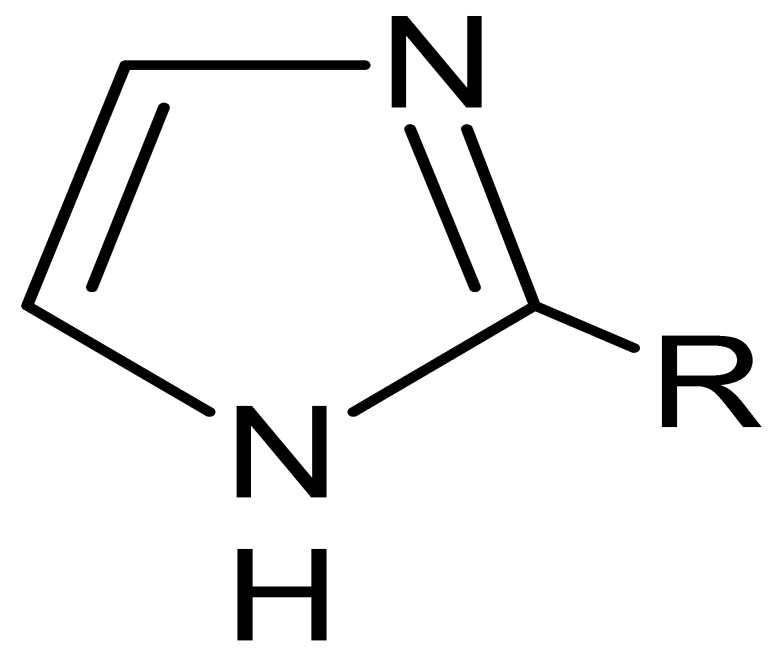	methyl CH3	**1**	8.05	46.0	281
	ethyl C2H5	**2**	8.00	48.0	285
	propyl C3H7	**3**	8.02	49.5	289
	butyl C4H9	**4**	8.00	52.0	292

**Table 2:** Average thickness and roughness of PIMs.

Membrane, no	1	2	3	4
Roughness, nm	3.55 ± 0.05	3.86 ± 0.05	4.12 ± 0.05	4.47 ± 0.05
Thickness, µm	26	28	27	30

Membrane: 2.6 cm^3^ o-NPPE/1 g CTA and 1.0 mol/dm^3^ carriers (**1**–**4**) (calculated on plasticizer).

**Table 3:** Kinetic parameters of transport by PIMs containing 2-alkylimidazoles and selectivity coefficient of Cu(II) ions.

2-Alkylimidazole	Metal Ion	J_0_, μmol/m^2^⋅s	S_Cu(II)/M(II)_ = J_0(Cu)_/J_0(M)_
1	Cu(II)	6.28 ± 0.01	Cu(II) > Zn(II) > Co(II) > Ni(II) 1.5 2.8 52.3
	Zn(II)	4.31 ± 0.01	
	Co(II)	2.26 ± 0.01	
	Ni(II)	0.12 ± 0.01	
2	Cu(II)	5.75 ± 0.01	Cu(II) > Zn(II) > Co(II) > Ni(II) 1.7 4.3 71.9
	Zn(II)	3.35 ± 0.01	
	Co(II)	1.33 ± 0.01	
	Ni(II)	0.08 ± 0.01	
3	Cu(II)	3.64 ± 0.01	Cu(II) > Zn(II) > Co(II) > Ni(II) 2.1 4.6 182
	Zn(II)	1.75 ± 0.01	
	Co(II)	0.79 ± 0.01	
	Ni(II)	0.02 ± 0.01	
4	Cu(II)	3.19 ± 0.01	Cu(II) > Zn(II) > Co(II) 2.6 6.9
	Zn(II)	1.22 ± 0.01	
	Co(II)	0.46 ± 0.01	
	Ni(II)	0.00 ± 0.01	

**Table 4:** Comparison of the stability constant values (log β) of Cu(II), Zn(II), Co(II), and Ni(II) complexes.

	Alkyl=	Cu(II) [62]	Zn(II) [63]	Co(II) [64]	Ni(II) [65]
1-Alkylimidazole	methyl	4.30	2.70	2.40	3.05
	ethyl	4.40	2.50	2.40	3.04
	propyl	4.25	2.62	2.38	3.06
	butyl	4.40	2.57	2.75	3.30
2-Alkylimidazole	methyl [66]	3.60	2.38	1.73	1.05
	ethyl [67]	3.35	1.80	1.49	0.65
	propyl [68]	3.11	1.12	0.57	0.24
	butyl [69]	2.86	0.74	0.23	-

Given values, the stability constants carry 5% tolerance.

**Table 5:** Diffusion coefficients (Do) of Cu(II), Zn(II), Co(II), and Ni(II) complexes with 2-alkylimidazoles and membrane diffusion resistance values (Δ_o_).

Carrier	Metal Ion	Δ_o_, s/m	D_o_, cm^2^/s
1	Cu(II)	114.1	2.38 × 10^−8^
	Zn(II)	153.8	4.21 × 10^−8^
	Co(II)	245.2	7.63 × 10^−8^
	Ni(II)	616.3	1.42 × 10^−10^
2	Cu(II)	136.5	1.17 × 10^−8^
	Zn(II)	187.2	3.96 × 10^−8^
	Co(II)	361.8	5.15 × 10^−8^
	Ni(II)	983.2	2.02 × 10^−11^
3	Cu(II)	206.4	3.13 × 10^−8^
	Zn(II)	415.6	4.68 × 10^−8^
	Co(II)	712.1	3.25 × 10^−9^
	Ni(II)	1083.7	4.13 × 10^−13^
4	Cu(II)	312.1	4.05 × 10^−9^
	Zn(II)	625.3	6.27 × 10^−9^
	Co(II)	947.5	2.01 × 10^−10^

**Table 6:** The copper (II) separation coefficients in relation to Zn(II), Co(II), and Ni(II) from their equimolar solutions after a 24 h transport across PIMs with alkylimidazoles.

Separation Coefficients Cu(II)/M(II)				
Carrier	Zn	Co	Ni	Ref.
1-hexylimidazole	4.3	39.7	46.9	[48]
1-hexyl-2-methylimidazole	3.9	24.8	59.1	[48]
1-decyl-4-methylimidazole	2.8	11.6	32.8	[48]
2-methylimidazole	1.5	2.8	52.3	this work
2-ethylimidazole	1.7	4.3	71.9	this work
2-propylimidazole	2.1	4.6	182	this work
2-butylimidazole	2.6	6.9		this work

**Table 7:** The values of Cu(II) recovery after a 24 h transport across PIMs doped with alkylimidazoles.

Carrier	Cu Recovery, %	Ref.
1-hexylimidazole	99.4	[48]
1-hexyl-2-methylimidazole	83.2	[48]
1-decyl-4-methylimidazole	93.0	[48]
2-methylimidazole	94.8	this work
2-ethylimidazole	94.0	this work
2-propylimidazole	90.0	this work
2-butylimidazole	87.0	this work

## Data Availability

The data used to support the findings of this study are available from the corresponding author upon request.
